# Pain-related somatosensory evoked potentials and functional brain magnetic resonance in the evaluation of neurologic recovery after cardiac arrest: a case study of three patients

**DOI:** 10.1186/1757-7241-20-22

**Published:** 2012-03-31

**Authors:** Paolo Zanatta, Simone Messerotti Benvenuti, Fabrizio Baldanzi, Matteo Bendini, Marsilio Saccavini, Wadih Tamari, Daniela Palomba, Enrico Bosco

**Affiliations:** 1 Department of Anesthesia and Intensive Care, Neurophysiology, Treviso Regional Hospital, Piazzale Ospedale 1, Treviso, 31100, Italy; 2 Department of General Psychology, University of Padova, Via Venezia 8, Padova, 35131, Italy; 3 Regional Project for the Reduction of Neurodysfunction after Cardiac Surgery and Neurosurgery, Improvement in Multimodality Neuromonitoring, Regione Veneto, Treviso Regional Hospital, Piazzale Ospedale 1, Treviso, 31100, Italy; 4 Department of Neuroradiology, Treviso Regional Hospital, Piazzale Ospedale 1, Treviso, 31100, Italy; 5 Department of Rehabilitation Medicine, Treviso Regional Hospital, Piazzale Ospedale 1, Treviso, 31100, Italy; 6 Department of Cardiac Surgery, Treviso Regional Hospital, Piazzale Ospedale 1, Treviso, 31100, Italy

**Keywords:** Electroencephalogram, Hypoxic-ischemic encephalopathy, Pain-related functional brain magnetic resonance, Pain-related somatosensory evoked potentials

## Abstract

This case series investigates whether painful electrical stimulation increases the early prognostic value of both somatosensory-evoked potentials and functional magnetic resonance imaging in comatose patients after cardiac arrest. Three single cases with hypoxic-ischemic encephalopathy were considered. A neurophysiological evaluation with an electroencephalogram and somatosensory-evoked potentials during increased electrical stimulation in both median nerves was performed within five days of cardiac arrest. Each patient also underwent a functional magnetic resonance imaging evaluation with the same neurophysiological protocol one month after cardiac arrest. One patient, who completely recovered, showed a middle latency component at a high intensity of stimulation and the activation of all brain areas involved in cerebral pain processing. One patient in a minimally conscious state only showed the cortical somatosensory response and the activation of the primary somatosensory cortex. The last patient, who was in a vegetative state, did not show primary somatosensory evoked potentials; only the activation of subcortical brain areas occurred. These preliminary findings suggest that the pain-related somatosensory evoked potentials performed to increase the prognosis of comatose patients after cardiac arrest are associated with regional brain activity showed by functional magnetic resonance imaging during median nerves electrical stimulation. More importantly, this cases report also suggests that somatosensory evoked potentials and functional magnetic resonance imaging during painful electrical stimulation may be sensitive and complementary methods to predict the neurological outcome in the acute phase of coma. Thus, pain-related somatosensory-evoked potentials may be a reliable and a cost-effective tool for planning the early diagnostic evaluation of comatose patients.

## Background

Early and accurate prognostic assessment of neurological functional outcomes in comatose patients after cardiac arrest is a relevant medical, ethical, and economic issue. It has been shown that beyond the Glasgow Coma Scale (GCS), a patient's pupil light reactivity, corneal reflexes, myoclonus status epilepticus, and serum neuron-specific enolase, short latency somatosensory-evoked potentials (SEPs) (N20/P25) improve the accuracy of neurological prognosis in comatose patients after cardiac arrest [1]. SEPs have shown high sensitivity and specificity in predicting poor outcomes. Indeed, that the bilateral disappearance of cortical N20/P25 is well-established to be associated with adverse outcomes such as death or survival in a vegetative state. Nonetheless, the presence of N20/P25 may not be sensitive enough to predict a good neurological outcome [2]. In fact, only the event-related evoked potentials (i.e., mismatched negativity and novelty P300), middle latency cortical somatosensory-evoked potentials (MLCEPs), and reactivity electroencephalogram (EEG) background have been associated with a favourable neurological prognosis [3-7].

In recent years, brain functional neuroimaging has been used in order to clarify the diagnosis of the vegetative state, suggesting that brain activation imaging may reflect awareness and/or cognition and provide reliable prognostic information [8,9]. However, given that this methodology is based on imagery and communication task paradigms, this approach is only feasible in the chronic phase of consciousness disorders. Moreover, studies using positron emission tomography have shown that minimally conscious state patients, compared to vegetative state patients, may show brain processing activation elicited by noxious electrical stimulation of the median nerves similar to that seen in healthy controls, suggesting a possible cortical processing of pain [10-12]. However, the intensity of electrical stimuli applied in vegetative patients (i.e., 14 mAmp) compared to healthy controls (i.e., 7 mAmp) does not seem adequate, in our opinion, to elicit a cortical response to pain. Indeed, the individual thresholds of sensitivity to pain can be higher in unresponsive brain injured patients compared to healthy subjects, as suggested by the unresponsiveness of blood pressure and heart rate during electrical stimulation in vegetative state patients [10]. Studies with functional magnetic resonance imaging (fMRI) showed the association between the activation of primary somatosensory cortex contralateral to somatosensory stimulation of the hand versus clinical measures of the level of consciousness during coma [13].

The possibility of exploring intraoperative pain monitoring for detecting the reactivity of both N20/P25 and MLCEPs during painful electrical stimulation of the median nerves in anaesthesia has been recently investigated [14]. This methodology can induce an increase in amplitude and decrease in latency of the N20/P25 and the appearance of MLCEPs, reflecting a more integrated cerebral processing of pain. More recent evidence has also revealed that MLCEPs elicited by painful electrical stimulation may be a sensitive method to predict the neurological outcome in comatose patients after cardiac arrest [15]. Specifically, patients who showed MLCEPs had a good neurological outcome, whereas the absence of MLCEPs was related to adverse neurological outcomes (i.e., minimally conscious state or vegetative state) in post-anoxic comatose patients. Based on these findings, assuming that the reactivity of SEPs to pain can reflect the integrity of the pain network of the nervous system, the main aim of the present case series was to evaluate whether pain-related N20/P25, MLCEPs, and fMRI may be reliable and accurate measures to predict the neurological prognosis of patients in the acute phase of post-anoxic comas. Accordingly, SEPs and fMRI were recorded during bilateral median nerves electrical stimulation in three post-anoxic comatose patients (i.e., cases 1, 2, and 3) who were enrolled in our previous study [15].

## Case presentation

### Cases

All three patients were admitted to the intensive care unit after cardiac arrest for acute coronary syndrome and were enrolled into this study. The patients were two males (i.e., patients 1 and 2) and one female (i.e., patient 3) aged 69, 71, and 64 years, respectively. Patients 2 and 3 had an out-of-hospital cardiac arrest. The etiopathogenesis of cardiac arrest was ventricular fibrillation for patient 1 and 2 and asystole for patient 3. All three patients were subjected to hypothermic treatment according to the International Guidelines for Resuscitation [16]. They had a resistant non-convulsive status epilepticus (NCSE) that was treated with a continuous intravenous infusion of midazolam (0.1 mg/kg/h), propofol (2 mg/kg/h), and two phenytoin boluses a day to maintain the blood drug level between 10-20 μg/ml. Approval for the present study was obtained from our Institution Ethical Committee. Moreover, informed consent was obtained from the next of kin or legal guardian of the patients. With respect to other sensitive stimulations, reactivity to painful stimuli is widely and routinely used to explore consciousness in clinical practice; the GCS represents the most commonly used clinical scale in exploring this issue [17]. Moreover, given that pain is an unpleasant experience that involves the conscious awareness of noxious sensations [18], this process is ineffective during coma and NCSE as our cases have shown. All the functional assessments were established during sedation for treating the NCSE or when the patients were in a deep comatose state (see Results section). For all of these reasons, in light of the actual knowledge, we consider the aims of our research to be ethically justified.

### Procedure

All of the patients underwent a neurophysiological, clinical, and fMRI evaluation according to the following time schedule: SEPs, EEG, and GCS were performed on day 2, after rewarming from moderate hypothermia (first evaluation); SEPs, EEG, GCS, and fMRI were performed after one month (second evaluation); GCS and the Levels of Cognitive Functioning scale (LCF) were performed after three months (third evaluation) [19]. With respect to other scales used for assessing recovery after brain injury, the LCF score responded better to our aim in grading the levels of cognitive capacity.

Neurophysiological and fMRI evaluations were performed with sedation in order to continue treating the NCSE and short acting muscle paralysing medications to reduce the artefacts induced by possible movements; a senior attending anaesthesiologist took care of the patients during the evaluations. During both neurophysiological and fMRI evaluations, the patients were bilaterally subjected to an increasing intensity of electrical stimulation of the median nerve in two steps: 10 and 50 mAmp. During each step, the following parameters were simultaneously recorded: the amplitude and the latency of N20/P25, EEG, heart rate (HR), and blood pressure (BP). HR and BP were measured as the most important signs of autonomic reactions elicited by the median nerve electrical stimulation.

During the fMRI evaluation, all patients were also subjected to 100 mAmp electrical stimulation due to the low sensitivity of 1.5 Tesla fMRI in viewing the activation of brain areas, especially in patients 2 and 3. At the one-month point, the neurophysiological evaluation was performed one hour before the fMRI. SEP recording during fMRI was not possible since scalp needle electrodes are incompatible with magnetic resonance imaging.

### Physiological recordings

The neurophysiological evaluation consisted of:

a) Bipolar eight-channel EEG recording using needle electrodes placed at the standard scalp sites (F3/F4-Cz, C3'/C4'-Cz, T3/T4-Cz, P3/P4-Cz); C3' and C4' were placed 2 cm posterior to the C3 and C4 positions of the 10/20 International System. The recording parameters of the EEG were as follows: sampling rate 250 Hz and hardware bandwidth 1 to 100 Hz, 1 Hz Low Frequency Filter (LFF), 40 Hz High Frequency Filter (HFF).

b) Three bilateral channels of somatosensory evoked potentials: (Fpz-C4'/C3'), (right Erb's point/left Erb's point-C4'/C3'), (Fpz-right Erb's point/left Erb's point). The recording parameters of SEP were 30 to 400 Hz for LFF and HFF, respectively, 10 msec/div, 100 sweeps; a further bandpass averaging filter (30-400 Hz) was applied. The sampling rate was 20 kHz and the hardware bandwidth was 1 to 4 kHz. The stimulus duration was 200 ms and the stimulus frequency was 3.3 Hz. Electrical stimulation was performed simultaneously, using needle electrodes placed on both wrists. The ground electrode was placed on the left shoulder for both EEG and SEPs. The electrode impedance was kept below 1 kΩ. EEGs and SEPs were recorded using the Eclipse Neurological Workstation-Axon System.

### Brain functional magnetic resonance imaging

A Siemens AVANTO 1.5 T MR system was used to perform fMRI with a 3D isovolumetric (1 × 1 × 1 mm) T1 sequence Turbo Gradient Echo (GRE) on the sagittal plane: Time Acquisition (TA) 5'32″, Time Repetition (TR) 9.5 ms, Time of Echo (TE) 4.76 ms, 160 slices, Matrix 256, field of view (FOV) 250, followed by blood oxygen level dependent fMRI with an echo planar sequence on the axial plane, TA 5'09″, TR 3820 ms, TE 50 ms, 36 slices, Matrix 128, FOV 230, thickness 3 mm, 80 measurements. An fMRI block design was used consisting of acquisition over 38.2 sec (10 blocks) of rest, followed by 38.2 sec (10 blocks) of electrical stimulation at 10 and 50 mAmp. Each design was repeated four times.

## Results

**Figure 1:**
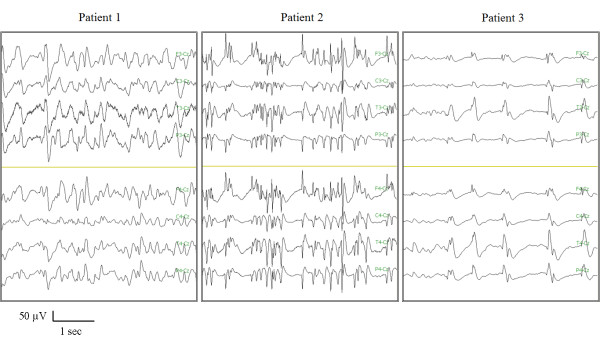
**Electroencephalogram of the three patients at the neurophysiological evaluation one month after cardiac arrest showing epileptic discharges for patients 1 and 2 and periodic spikes for Patient 3**.

The neurophysiological evaluation, fMRI, GCS, and LCF at three months were different for each case. Each patient had a GCS of 3 at day 2 after cardiac arrest. The EEG at day 2 showed epileptic discharges consistent with NCSE in all patients; they were also detected at 1 month with the exception of patient 3 who showed periodic spikes (Figure 1). During the electrical stimulation, all the patients showed an increase in BP at day 2 and at one month. No variation was detected in HR.

### Case 1

He recovered consciousness after 50 days corresponding to the resolution of the NCSE. He was discharged from the intensive care unit to a rehabilitation unit after 43 days. At three months, he showed good neurological recovery, with some residual polyneuropathy of the lower limbs and an LCF of 8. The GCS scale was 5 (E1VtM4) and 15 (E4V5M6) at one and three months, respectively. The neurophysiological evaluation performed at day 2 and one month showed that increasing electrical stimulation induced the appearance of MLCEPs with higher amplitude in the right hemisphere, peaking at 50-60 ms after stimulus onset. The same stimulation increased the amplitude and reduced the latency of the N20/P25, as reported in Table 1.

**Table 1:** Differences of measures between 10-50 mAmp electrical stimulations in Patient 1 at day 2 and one month after cardiac arrest

Patient 1	First Evaluation (day 2)		Second Evaluation (1 month)	
**Measures**	**10 mAmp**	**50 mAmp**	**10 mAmp**	**50 mAmp**

Left N20 amplitude (μV)	2.1	3.6	2.4	3.6

Right N20 amplitude (μV)	2.9	4.4	2.5	4

Left N20 latency (ms)	23.4	23.2	23.7	22.5

Right N20 latency (ms)	22.5	22.3	23.3	22.8

HR (bpm), Mean	129	129	99	110

BP (mmHg)	119/85	128/91	115/75	142/88

GCS*	E1VtM1		E1VtM4	

HR: heart rate.

BP: blood pressure

*GCS: Glasgow Coma Scale

**Figure 2:**
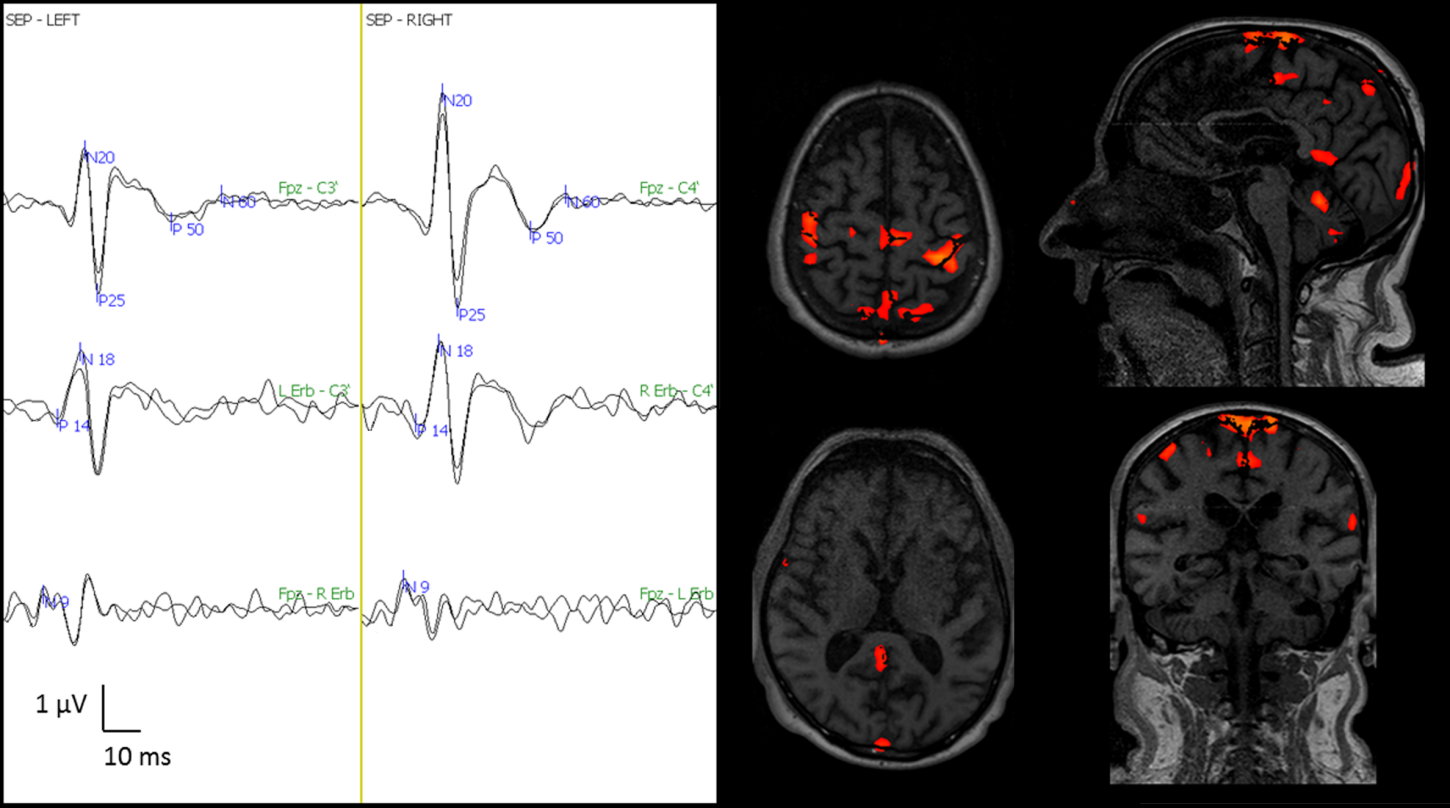
**Pain-related SEP and fMRI one month after cardiac arrest**. Left side: Three bilateral SEP channels: Fpz - C3'/C4', left Erb's/right Erb's - C3'/C4', Fpz- right Erb's/left Erb's. Note the appearance of MLCEP (P50/N60), which is more evident on the right side. Right side: Four fMRI scans at 99% sensitivity during the 50 mAmp electrical stimulation. Note the activation of bilateral primary, secondary, motor, posterior cingulated, and cerebellar cortex.

**Figure 3:**
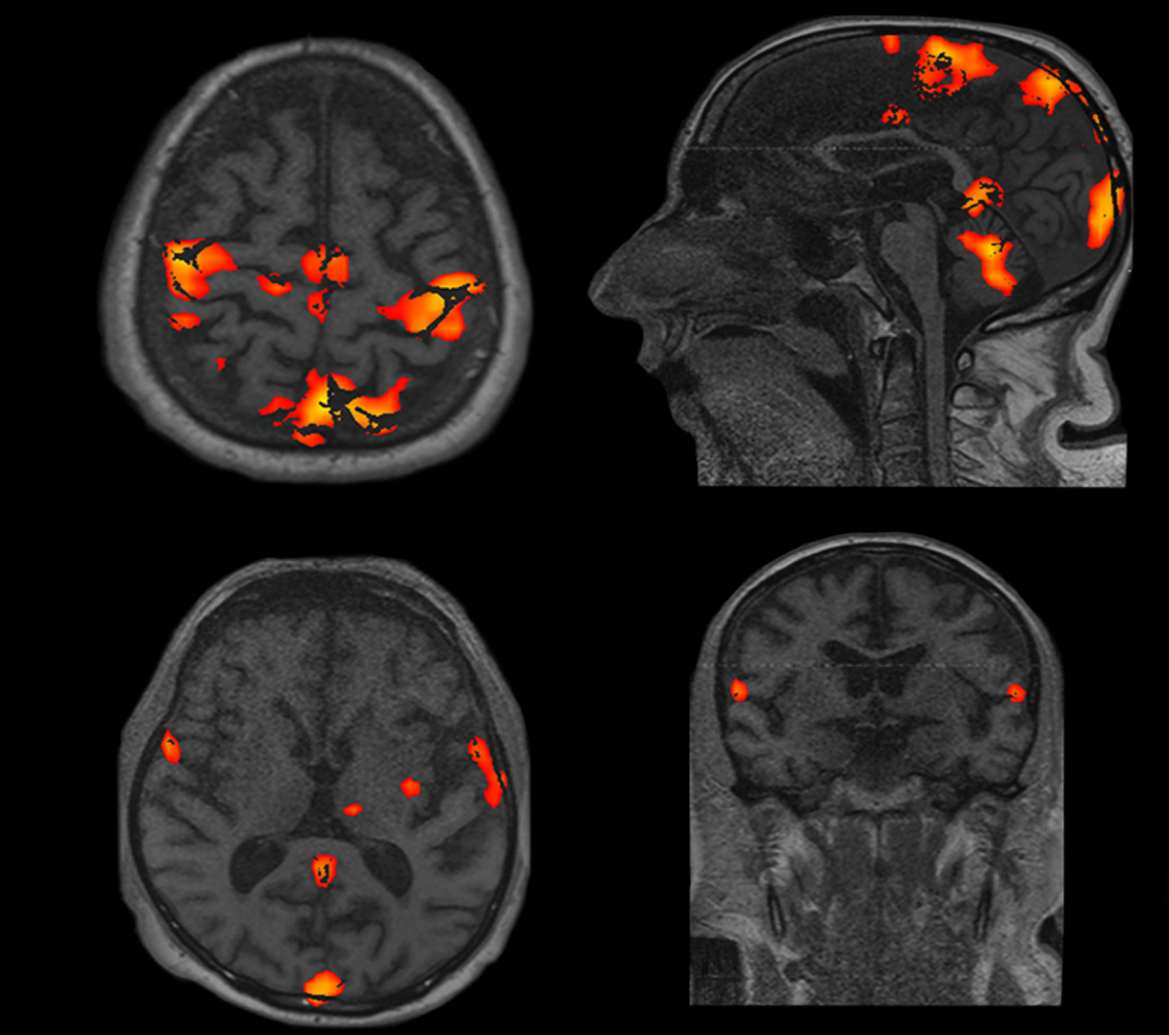
**fMRI with 99% sensitivity during the 100 mAmp electrical stimulation one month after cardiac arrest showed the activation of the left thalamus, left insula, and cerebellar cortex and a more intense activation of the brain areas previously described in Figure 2**.

The fMRI with 99% sensitivity during the 50 mAmp electrical stimulation showed activation of the brain area involved in the processing of painful stimulation (i.e., primary and secondary somatosensory areas, posterior cingulate), as shown in Figure 2; the primary motor cortex was still activated. The fMRI with 99% sensitivity during the 100 mAmp electrical stimulation showed activation of the left thalamus, left insula, and cerebellar cortex and a more intense activation of the brain areas previously described (Figure 3). After fMRI evaluation, the patient was subjected to a continuous infusion of sodium thiopental for three days in order to obtain an electroencephalographic burst suppression pattern for treating NCSE.

### Case 2

After three months, patient 2 was minimally conscious [20]. He showed an affective behaviour and a response to painful stimulation with modification of facial expression and lacrimation. He did not perform requested commands. He displayed spastic palsy to a higher extent in the lower limbs. The LCF scale was 3 and the GCS was 8 (E4VtM4). He was discharged from the intensive care unit to a rehabilitation unit after 20 days. After one month, the GCS scale was 3 (E2VtM1). Increasing electrical stimulation enhanced the amplitude and reduced the latency of the N20/P25 after one month while MLCEPs did not occur, as reported in Table 2. At day 2, the neurophysiological evaluation showed an appearance of the first N20/P25 at 50 mAmp but not at 10 mAmp.

**Table 2:** Differences of measures between 10-50 mAmp electrical stimulations in Patient 2 at day 2 and one month after cardiac arrest

Patient 2	First Evaluation (day 2)		Second Evaluation (1 month)	
**Measures**	**10 mAmp**	**50 mAmp**	**10 mAmp**	**50 mAmp**

Left N20 amplitude (μV)	0	0.45	1.3	1.8

Right N20 amplitude (μV)	0	0.33	0.74	1.6

Left N20 latency (ms)	-	28	24.8	23.8

Right N20 latency (ms)	-	26.9	24.8	23.4

HR (bpm), Mean	66	68	55	56

BP (mmHg)	120/60	135/64	130/70	150/60

GCS *	E1VtM1		E1VtM1	

HR: heart rate.

BP: blood pressure

*GCS: Glasgow Coma Scale

**Figure 4:**
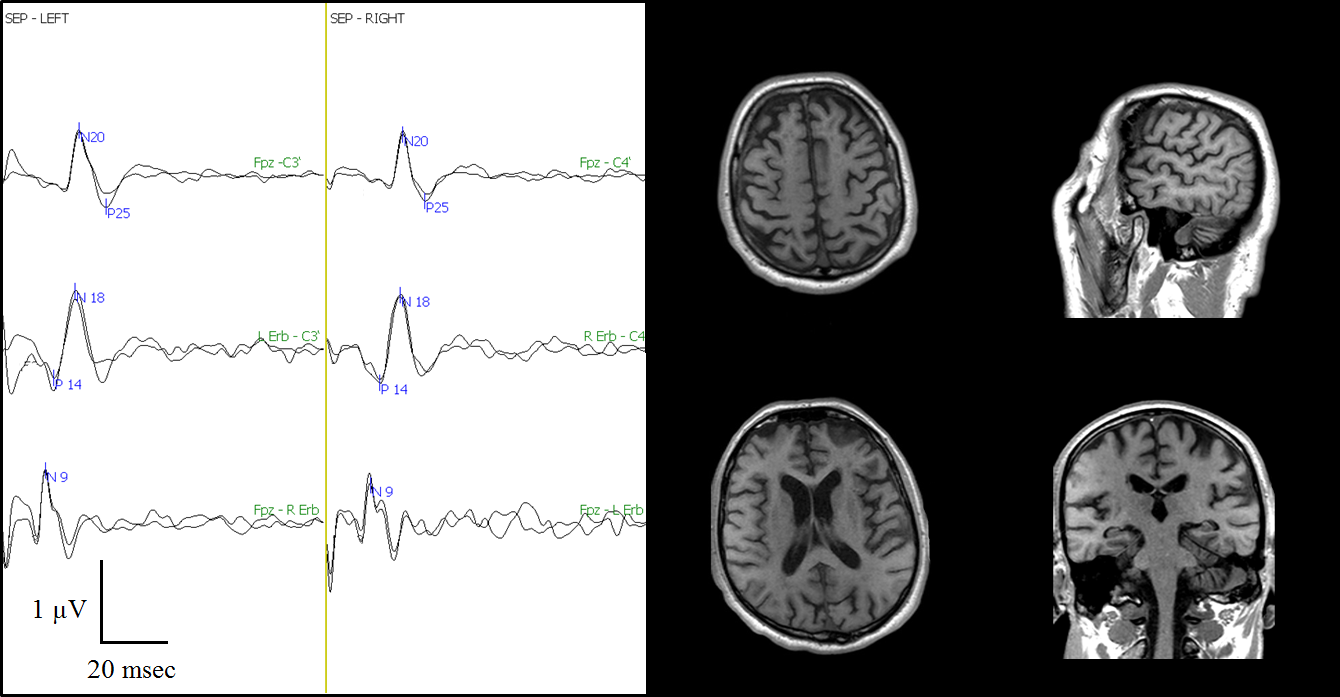
**Pain-related SEP and fMRI one month after cardiac arrest**. Left side: Three bilateral SEP channels: Fpz - C3'/C4', left Erb's/right Erb's - C3'/C4', Fpz - right Erb's/left Erb's. Note the presence of the N20/P25 cortical potential. Right side: Four fMRI scans at 99% sensitivity during the 50 mAmp electrical stimulation. No single brain area was activated at 50 mAmp.

**Figure 5:**
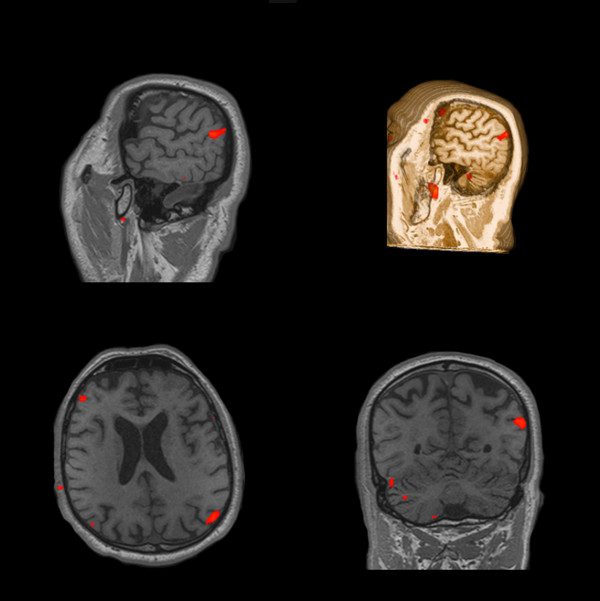
**fMRI with 60% sensitivity during the 100 mAmp electrical stimulation one month after cardiac arrest showed a prevalent activation of the right primary somatosensory cortex**.

The fMRI with 99% sensitivity did not show any activation of the brain cortex during the 50 mAmp stimulation (Figure 4), while a prevalent activation of the right somatosensory cortex during the 100 mAmp stimulation occurred with 60% sensitivity fMRI (Figure 5).

**Figure 6:**
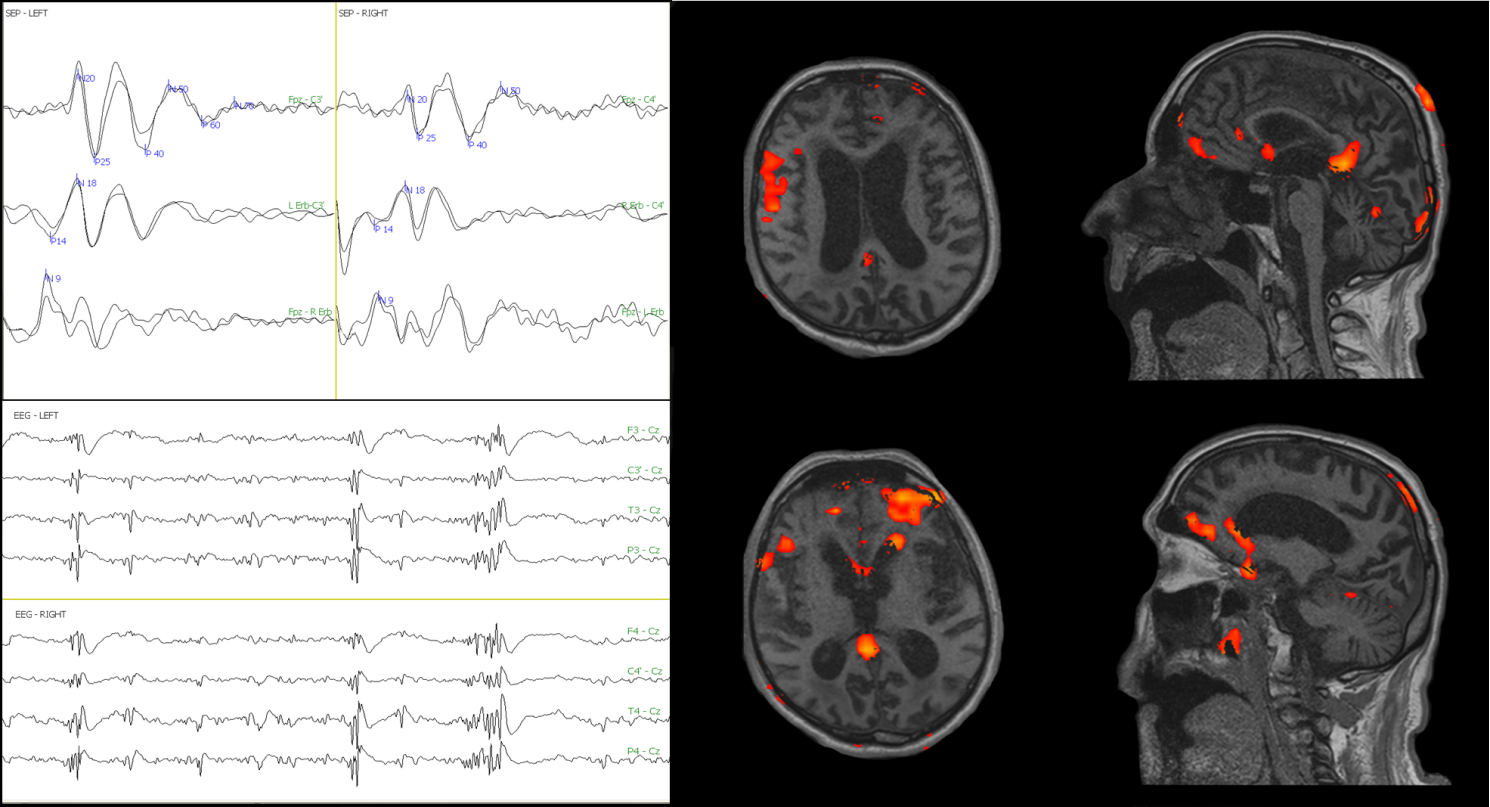
**Pain-related SEP and fMRI six months after cardiac arrest**. Left side: Three bilateral SEP channels: Fpz - C3'/C4', left Erb's/right Erb's - C3'/C4', Fpz - right Erb's/left Erb's. Note the appearance of bilateral MLCEP (40-50 ms) and left MLCEP (60-70 ms) and NCSE in the EEG traces. Right side: Four fMRI scans at 99% sensitivity during the 50 mAmp electrical stimulation. Note the activation of the secondary somatosensory, posterior-anterior cingulate, frontal, cerebellar cortex, and amygdale.

Clinical, neurophysiological, and fMRI evaluations were also performed after six months. The clinical evaluation (i.e., the LCF and GCS scores) did not change compared to the evaluation after three months. Patient 2 showed an EEG pattern consistent with NCSE at the six-month neurophysiological evaluation. In this patient, increasing electrical stimulation induced the bilateral appearance of MLCEPs, peaking at 40-50 ms and left hemisphere 60-70 ms after stimulus onset. The same stimulation also increased the amplitude and reduced the latency of the N20/P25 (Table 3). The fMRI with 99% sensitivity during the 50 mAmp electrical painful stimulation showed an activation of the brain area involved in processing pain and emotion (i.e., the secondary somatosensory, posterior-anterior cingulate, frontal cortex, cerebellar cortex and amygdala, as shown in Figure 6).

**Table 3:** Differences of measures between 10-50 mAmp electrical stimulations in Patient 2 six months after cardiac arrest

Patient 2	6-Month Evaluation	
**Measures**	**10 mAmp**	**50 mAmp**

Left N20 amplitude (μV)	2.2	2.6

Right N20 amplitude (μV)	3.9	5

Left N20 latency (ms)	21.8	21.7

Right N20 latency (ms)	23	22.7

HR (bpm), Mean	59	62

BP (mmHg)	135/66	157/72

GCS *	E4VtM4	

HR: heart rate.

BP: blood pressure

*GCS: Glasgow Coma Scale

### Case 3

After three months, Case 3 was in a vegetative state [21]. She did not show any signs of conscious perception regarding the different types of stimulation, including pain. The LCF scale was 2 and GCS was 7 (E3VtM4). She was discharged from the intensive care unit to a rehabilitation unit after 23 days. The GCS scale after one month was 5 (E1VtM4). Painful cutaneous stimulation induced slight opening of the eyes with flexion of the right arm.

**Figure 7:**
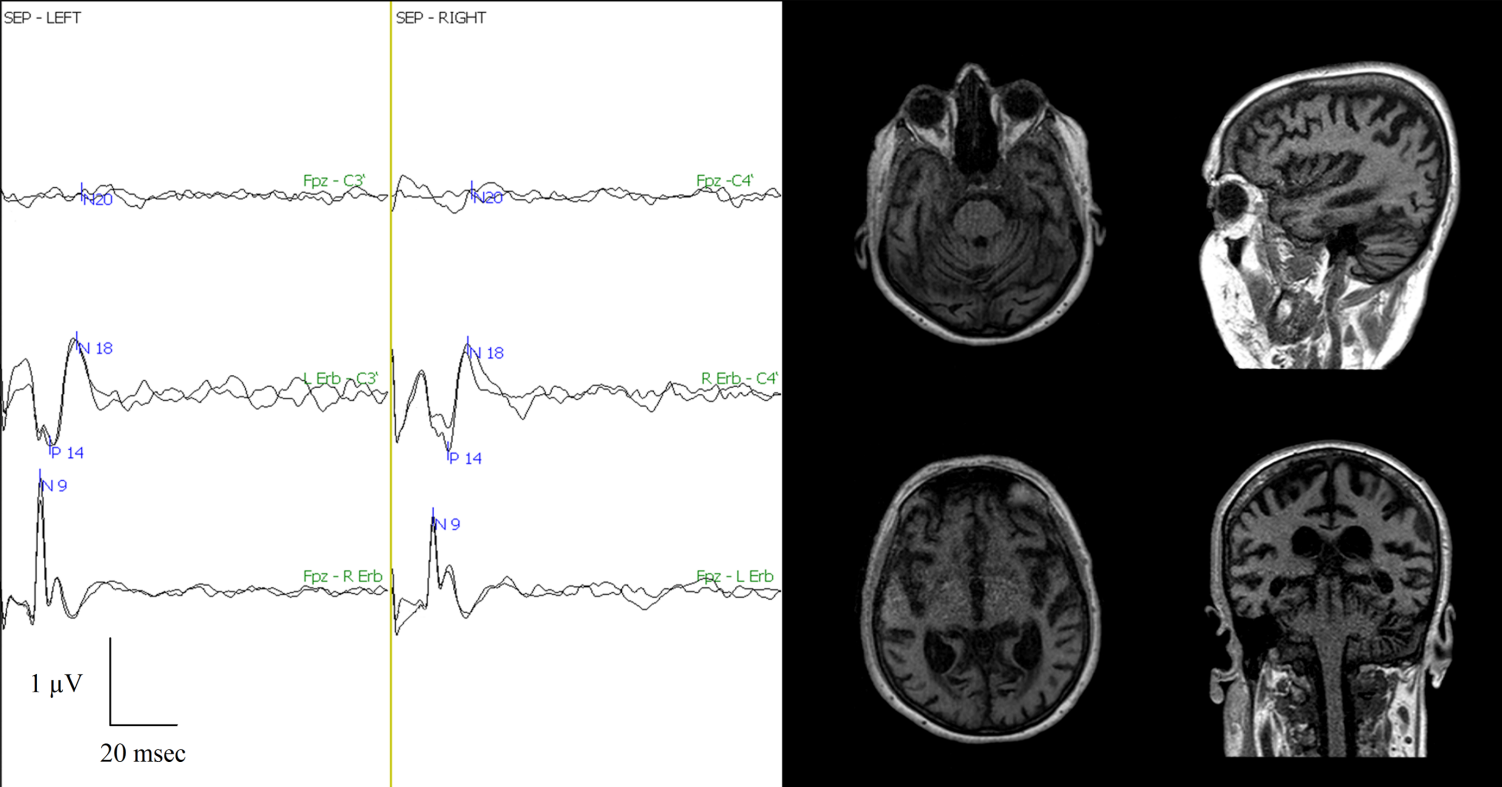
**Pain-related SEP and fMRI one month after cardiac arrest**. Left side: Three bilateral SEP channels: Fpz - C3'/C4', left Erb's/right Erb's - C3'/C4', Fpz - right Erb's/left Erb's. Note the absence of N20/P25. Right side: Four fMRI scans at 99% sensitivity during the 50 mAmp electrical stimulation. No single brain area was activated at 50 mAmp.

**Figure 8:**
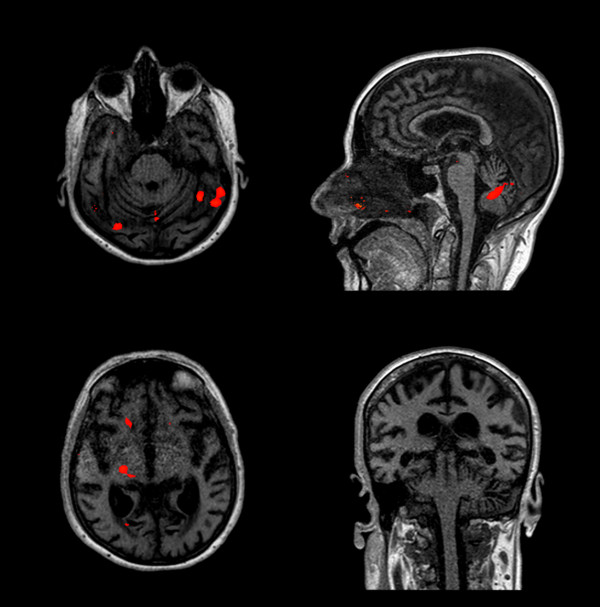
**fMRI with 60% sensitivity during the 100 mAmp electrical stimulation one month after cardiac arrest showed the activation of the right thalamus and left cerebellar cortex**.

The neurophysiological evaluation at day 2 and at one month showed an absent N20/P25, even with high-intensity electrical stimulation (Table 4). The fMRI, with 99% sensitivity at 50 mAmp, did not show any activation of the brain areas associated with pain perception (Figure 7). After reducing the sensitivity to 60% during the 100 mAmp electrical stimulation, activation of the right thalamus and left cerebellum was shown (Figure 8).

**Table 4:** Differences of measures between 10-50 mAmp electrical stimulations in Patient 3 at day 2 and one month after cardiac arrest

Patient 3	First Evaluation (day 2)		Second Evaluation (1 month)	
**Measures**	**10 mAmp**	**50 mAmp**	**10 mAmp**	**50 mAmp**

Left N20 amplitude (μV)	0	0	0	0

Right N20 amplitude (μV)	0	0	0	0

Left N20 latency (ms)	-	-	-	-

Right N20 latency (ms)	-	-	-	-

HR (bpm), Mean	90	98	55	56

BP (mmHg)	130/80	147/86	135/66	149/70

GCS *	E1VtM1		E2VtM4	

HR: heart rate.

BP: blood pressure

*GCS: Glasgow Coma Scale

Given that there was no bilateral N20/P25, and therefore no cortical perception at day 2, the neurophysiological and fMRI evaluation after one month was only established with muscle relaxant medications; no sedative was given to the patient at this time in order to not depress possible brain cortical response(s). All fMRI, neurophysiological, and neurovegetative data are reported along with LCF after three months in Table 5.

**Table 5:** Relationship between outcome, neurohysiological, fMRI, and vegetative reactivity during painful electrical stimulation at one month after cardiac arrest

Patient	Outcome	fMRI	MLCEP	N20/P25 Reactivity	BP Reactivity
1	Good	Cortical	yes	yes	yes

2	MCS	SI	no	yes	yes

3	VS	Sub-cortical	no	no	yes

MLCEP: middle latency cortical evoked potential

BP: blood pressure

MCS: minimally conscious state

VS: vegetative state

SI: primary somatosensory cortex

## Discussion

This case series examined whether SEPs and fMRI during a high intensity electrical stimulation in both median nerves may be accurate measures to assess the neurological outcome of patients in the acute phase of hypoxic-ischemic encephalopathy.

The current three cases were included in our previous study (i.e., patients' number 2, 5, and 8 for cases 1, 2, and 3, respectively), in which we provided preliminary evidence for the predictive value of MLCEPs in post-anoxic comatose patients [15]. Specifically, we found that the presence of MLCEPs was related to good neurological outcome, whereas the absence of MLCEPs was indicative of adverse neurological outcome (i.e., minimally conscious state or vegetative state) of patients in the acute phase of coma. The present case series extends our recent study by showing, on the one hand, the regional brain activity elicited by electrical stimulation of median nerves and, on the other hand, the relationship between the neurophysiological (i.e., SEPs) and neuroimaging (i.e., fMRI) responses to noxious stimulation in post-anoxic comatose patients.

The appearance of the MLCEPs in Case 1 was in agreement with studies showing a cortical biphasic response in the opercular cortex (i.e., secondary somatosensory and insular cortex) and with the functional characterisation of nociceptive areas near the lateral sulcus, as also suggested by fMRI data [22,23]. Indeed, the appearance of MLCEPs seems to be an expression of the activation of the brain areas involved in processing painful stimuli [14]. The activation of the primary motor cortex can be considered a useful prognostic measure indicating residual functional organization of the brain [24]. MLCEPs may be an accurate neurophysiological measure for the prognosis of good neurological outcome given that it represents a sensory activation beyond the primary cortex and is an expression of thalamo-cortical and cortico-cortical networks [3,6]. Similar brain activation elicited by other stimulation (i.e., visual or acoustic stimuli) has been associated with recovery from unconsciousness [8,9]. In our cases (i.e., patients 2 and 3) the lack of MLCEPs after one month during intense electrical stimulation suggests the absence of the brain connectivity in comatose patients, which, in turn, may be associated with an adverse neurological outcome.

This case series showed that the cortical N20/P25 along with MLCEPs reactivity to painful electrical stimulation can also be a useful method in detecting brain cortical availability and brain pain networks in comatose patients. This technique may detect the activation of brain tissue still alive but quiescent in the ischemic penumbra. Indeed, during the first evaluation in Case 2, only 50 mAmp of stimulation elicited a cortical response. This result agrees with the fact that, within 24 hours after the restoration of spontaneous circulation, there is improvement of the N20/P25, reflecting a reduction in the ischemic penumbra [25]. We suggest that N20/P25 could differentiate between minimally conscious and vegetative states since it reflects the activation of more areas of the brain cortex. N20/P25 may represent an early neurophysiological measure of possible recovery toward a minimally conscious state. Indeed, in agreement with previous neuroimaging studies, the neurophysiological evaluation of Case 2 after six months showed a reactivity of N20/P25 and MLCEPs associated with cerebral activation elicited by processing of pain during fMRI [11,12]. Our study suggests that the different latencies of the MLCEPs may be associated with the activation of different brain area in fMRI. Results from cases 1 and 2 also suggest that MLCEPs may represent the integrity of the fronto-parietal network involved in the mechanism of the contents of consciousness (awareness) [26]. These findings are in line with recent evidence showing that cortical connectivity and consciousness recovery can be assessed in patients surviving after severe brain injury [27].

In Case 3, the absence of N20/P25 corresponds to absent activation of the primary brain cortex; these data are in agreement with a vegetative state condition. It is interesting to note that the GCS motor response to pain (flexion of the right arm) is in agreement with the activation of the right thalamus and left cerebellum, even with the 60% sensitivity of fMRI. Although more data are necessary to establish the brain networks involved in pain processing, this case suggests a subcortical-like network in the integration of painful stimuli.

Although NCSE is known as an independent negative outcome predictor [28], NCSE did not interfere with the detection of MLCEPs in these three cases. This latter finding is in line with more recent evidence showing a lack of correlation between the EEG rhythms, somatosensory evoked cortical response, and good neurological outcome [29,30]. Indeed, the recovery of consciousness in patients with preserved SEPs may depend on the time of recovery of a normal EEG rhythm [30].

The current methodology may represent a dynamic test of brain availability similar to the stress echo and magnetic resonance imaging for the myocardium's functional reserve and the hibernating myocardium, respectively [31]. We suggest that this method may be considered a sort of neurophysiological GCS with which the physician evaluates the brain's reactivity to painful stimulation.

Moreover, our case series also showed potential for treating NCSE in the early phase after cardiac arrest in patients who only present with the N20/P25 cortical (Case 2); this pharmacological treatment along with therapeutic hypothermia [29] may be effective in restoring the brain network involved in control of the levels of consciousness (arousal).

Our case reports provide further evidence for the role of a multimodality approach with SEPs and EEG for predicting the prognosis of post anoxic coma. Indeed, SEPs and EEG can explore the neurophysiological basis of awareness and arousal, respectively; our findings also suggest that the pain-related SEPs may increase the efficacy in predicting the good neurological recovery in comatose patients. For all of these reasons, we suggest that the combination of SEPs and EEG should have a wider application in clinical practice [32].

Some limitations must be recognised in interpreting our data. First, it is important to note that the data presented in the current study are preliminary and need to be replicated by future rigorous research. Specifically, a larger number of patients, control groups, and long-term follow-ups should be carried out in order to fully estimate the effectiveness of pain-related SEPs and fMRI in predicting the neurological outcome of patients with hypoxic-ischemic encephalopathy. Second, an MRI with a magnetic field strength greater than 1.5 T should be used in order to accurately detect the activation of cortical brain areas with both 10 and 50 mAmp of electrical stimulation. However, the neuroimaging results obtained during the 100 mAmp stimulation performed to enhance the sensitivity of fMRI provide further evidence for the association between the neuroimaging and neurophysiological findings. Third, although laser-evoked potentials could be used, we considered the electrical pain stimulation to be a more accurate method for investigating the activation of the pain fibres in the difficult scenario of the intensive care settings. Finally, to reduce the time spent on the analysis and to improve the signal-to-noise ratio, it may be useful to treat patients with a muscle relaxant.

## Conclusions

The present cases report, albeit preliminary, suggests that somatosensory evoked potentials and functional magnetic resonance imaging performed along with pain-related methods may be useful measures for providing prognostic information in patients with hypoxic-ischemic encephalopathy. Along the same line of reasoning, this small case series shows cortical reactivity to nociceptive stimulation may improve the diagnostic efficacy of the standard neurological examination in comatose patients. This cases report also contributes to the growing interest in combining neurophysiological and neuroimaging techniques in order to evaluate neurological outcomes in comatose patients. Finally, the current case series suggests the potential usefulness of an integrated multidisciplinary approach between neurophysiologists, intensivists, neuroradiologists, and rehabilitation physicians.

## Abbreviations

BP: Blood pressure; EEG: Electroencephalogram; fMRI: Functional magnetic resonance imaging; FOV: Field of view; GCS: Glasgow coma scale; GRE: Turbo gradient echo; HFF: High frequency filter; HR: Heart rate; LCF: Levels of cognitive functioning scale; LFF: Low frequency filter; MLCEPs: Middle latency cortical evoked potentials; MRI: Magnetic resonance imaging; NCSE: Non-convulsive status epilepticus; SI: Primary somatosensory cortex; SEPs: Somatosensory evoked potentials; TA: Time acquisition; TE: Time of echo; TR: Time repetition.

## Written informed consent

Written informed consent was obtained from the next of kin or legal guardian of each patient.

## Competing interests

The authors declare that they have no competing interests.

## Authors' contributions

PZ made the main substantial contribution to the idea, conception and design, acquisition of data, analysis and interpretation of data, and was mainly involved in drafting the manuscript and in its critical revision. SMB was involved in the analyses and interpretation of data and in drafting and critically revising the manuscript. FB participated in the neurophysiological recordings, collected the data, and contributed the graphic artwork. MB carried out the fMRI acquisitions. DP and WT provided useful comments. MS performed the clinical evaluations three and six months after cardiac arrest. EB was involved in the conception and design of the study and in its critical revision. All of the authors read and approved the final manuscript.
